# How the mosquito’s carbon dioxide sense gets an internal boost

**DOI:** 10.1371/journal.pbio.3003958

**Published:** 2026-09-11

**Authors:** Chih-Ying Su

**Affiliations:** 1 Department of Neurobiology, University of California San Diego, La Jolla, California, United States of America; 2 Institute of Bio-Architecture and Bio-Interactions (IBABI), Shenzhen Medical Academy of Research and Translation (SMART), Shenzhen, Guangdong, China

## Abstract

Mosquitoes use carbon dioxide as a critical cue to sense if humans are nearby. This Primer explores a new study in PLOS Biology that reveals that carbon dioxide-sensing neurons in mosquitoes can amplify each other’s signals, creating a neural echo chamber that may explain the remarkablesensitivity of mosquitoes to human presence.

## Teaser

Mosquitoes use CO_2_ as a critical cue to sense if humans are nearby. A new study in *PLOS Biology* reveals that CO_2_-sensing neurons in mosquitoes can amplify each other’s signals, creating a neural echo chamber that may explain the remarkable sensitivity of mosquitoes to human presence.

Mosquitoes are the deadliest animals on Earth, and *Aedes aegypti* is among the most dangerous. Its extraordinary ability to find us and transmit diseases such as dengue, Zika, chikungunya, and yellow fever relies on carbon dioxide (CO_2_). While other odors, heat, and visual cues contribute, CO_2_ plays a special role, activating the mosquito and sensitizing it to our presence [1]. How a sensory system achieves such a powerful behavioral response from a single chemical cue has been a long-standing question.

**Fig 1:**
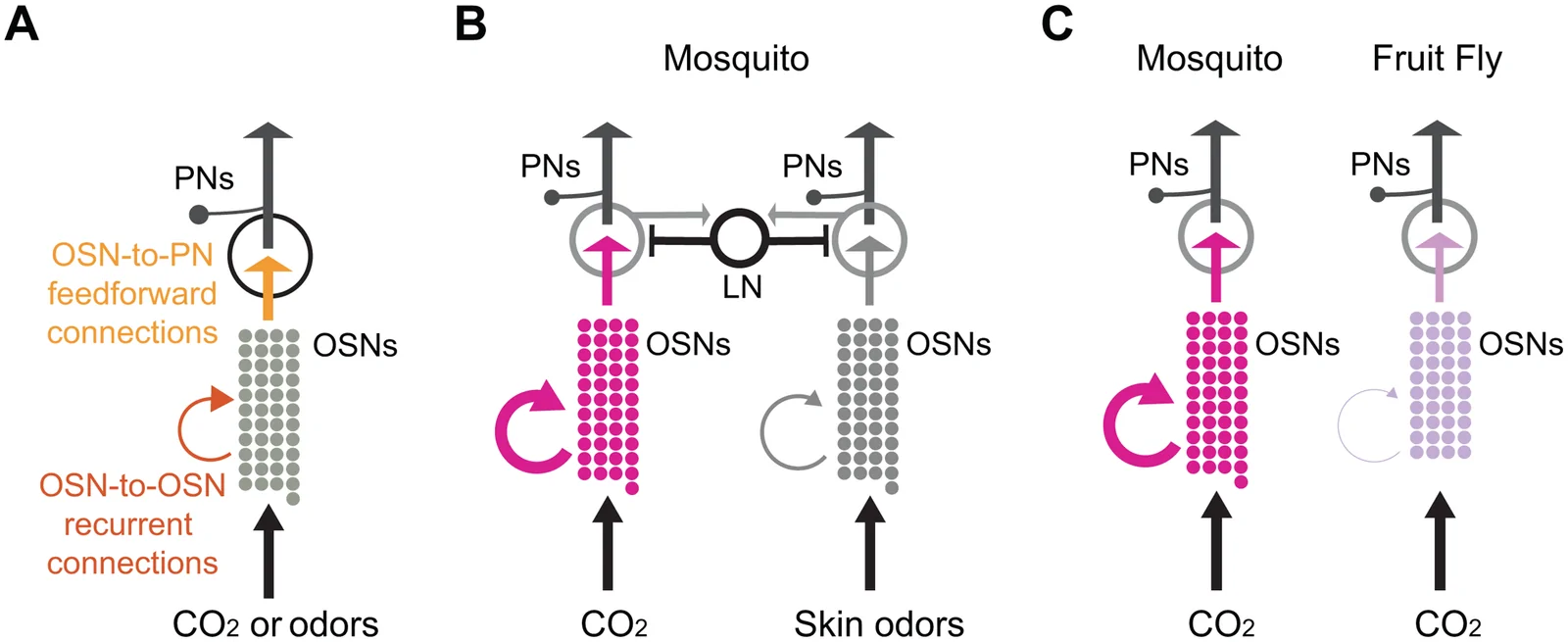
Comparative circuit architecture of CO_2_- and odor-sensing OSNs in mosquitoes and flies. **(A)** Insect olfactory sensory neurons (OSNs) expressing the same receptor converge onto a single glomerulus (circle) in the antennal lobe, where they synapse onto projection neurons (PNs). This study focuses on two circuit motifs: recurrent OSN-to-OSN connections (for signal amplification) and feedforward OSN-to-PN connections (for information relay). **(B)** OSNs also synapse onto inhibitory local neurons (LNs) When multiple OSNs are co-activated, they recruit local neurons to mediate global lateral inhibition for divisive normalization. In mosquitoes, both CO_2_- and odor-sensing OSNs express GABA receptors (likely at their axonal terminals), rendering them susceptible to this inhibition. Only CO_2_-sensing OSNs exhibit strong recurrent connections, which amplify their output signal and protect it from divisive normalization. **(C)** Strong recurrent connectivity is observed among mosquito CO_2_-sensing OSNs, but is absent in either CO_2_- or odor-sensing OSNs of fruit flies. Images adapted from Bao and colleagues [5].

In the insect olfactory system, sensory information passes from olfactory sensory neurons (OSNs) to the antennal lobe, where feedforward excitatory synapses relay signals to projection neurons and onward to higher brain centers (Fig 1A). A mosquito encountering hosts detects not only CO_2_ but also a complex blend of skin odors. When multiple OSNs are co-activated, they engage inhibitory local neurons that project back to OSN axonal terminals, where they dampen synaptic output via GABA receptors expressed in OSNs (Fig 1B). This negative feedback suppresses most co-active OSNs—a divisive normalization mechanism that scales down incoming signals to preserve the system’s dynamic range [2–4]. In essence, by turning down overall sensitivity, lateral inhibition ensures that subtle differences between scents remain discernable while keeping the system from saturating, even at high odor intensities.

That presents a potential problem: if divisive normalization dampens CO_2_ signal just as much as background odors, the mosquito may fail to become sufficiently aroused to seek a host. In a new study in PLOS Biology, Bao, Alford and colleagues use a high-resolution connectome to reveal how the CO_2_ circuit overcomes this conundrum [5].

Using automated serial-section transmission electron microscopy, the authors reconstructed the circuitry of the three glomeruli innervated by OSNs from the maxillary palp, including the CO_2_-responsive glomerulus and two adjacent glomeruli that receive input from odor-sensing OSNs. Their findings reveal a striking departure from the standard olfactory circuit architecture. CO_2_-sensitive OSNs form an unexpectedly high number of recurrent or reciprocal synapses with one another, compared to their odor-sensing counterparts (Fig 1B). In fact, these CO_2_ neurons send more signals back to each other than they send forward to their target projection neurons. Think of it like an echo chamber: the CO_2_ message bounces among the same neurons before being passed up the chain. This built-in “echo” creates a circuit that is specifically wired to amplify even a faint CO_2_ whiff—turning a subtle cue into a strong, resounding alert.

To understand the functional significance of this echo chamber, the team built a computational model. Their simulations suggest that recurrent connectivity is not an anatomical curiosity but a computational strategy for reliable CO_2_ detection in a noisy olfactory world. Without amplification, weak signals would be lost among background odors; with it, detection becomes robust. Yet the model also revealed a trade-off: recurrent amplification increases sensitivity but also raises the risk of false alarms. This suggests the mosquito’s brain is wired to prioritize detecting CO_2_—an ethologically critical cue for survival—over discrimination.

The study’s significance extends beyond a simple wiring diagram. The authors identified a potential anatomical specialization that underpins this recurrent signaling: the presence of ribbon-like presynaptic structures at these synapses. These structures, which are hallmarks of sustained and graded synaptic vesicle release in vertebrate sensory systems [6], have not previously been identified in invertebrates. Their presence at the synapses between mosquito CO_2_ OSNs suggests a convergently evolved mechanism for robust synaptic transmission. The authors also provide transcriptomic evidence that these OSNs express nicotinic acetylcholine receptors, supporting the possibility that these recurrent connections are excitatory.

What are the broader implications? First, circuit logic is not dictated by odorant or receptor identity alone. Flies and mosquitoes both detect CO_2_ using orthologous receptors [7], yet their circuit architectures diverge. Unlike the mosquito CO_2_ glomerulus, fly’s CO_2_ glomerulus (or odor-sensing glomeruli) lacks dense recurrent connectivity (Fig 1C). This likely reflects their opposing ethological roles: flies interpret CO_2_ detected by ab1C OSNs as an alarm cue and avoid it, whereas mosquitoes interpret the exact same gas as an arousal cue, using it to zero in on their host [7]. Morphology reinforces this divide: mosquito CO_2_-sensing neurons have roughly 6-fold larger sensory surfaces than the fly counterparts [8,9], potentially improving CO_2_ sensitivity. In other words, flies and mosquitoes detect the same CO_2_ cue, yet their CO_2_-sensing neurons are distinctly wired and shaped, each reflecting the specific ethological demands of its species.

Remarkably, in flies and (likely) mosquitoes, the CO_2_-sensing glomeruli manage to escape divisive normalization—but they do so through entirely different means. Flies achieve this by downregulating GABA receptors on their CO_2_-sensing glomerulus, effectively shielding it from lateral inhibition [4]. Mosquitoes, by contrast, maintain normal levels of GABA receptor expression and likely remain susceptible to lateral inhibition, yet recurrent connections are expected to amplify the signal to circumvent divisive normalization. That the very same glomerular target avoids the same constraint via such divergent strategies—receptor tuning versus circuit engineering—is striking and warrants future comparative investigation into their physiological significance.

Looking forward, this work opens several new avenues. The most immediate priority is functional validation: despite the greater technical challenge in mosquitoes compared to fruit flies, experiments should confirm that the CO_2_-sensing glomerulus evades divisive normalization and test whether disrupting recurrent connectivity compromises CO_2_ detection. At the circuit level, a whole-brain connectome would illuminate how CO_2_ integrates with other host cues. Ultimately, comparative connectomics across sexes and mosquito species with different host preferences could reveal whether recurrent connection is broadly conserved among blood-seeking mosquitoes.

For now, Bao, Alford and colleagues have provided a connectomic blueprint of how a deadly mosquito detects our presence with exquisite sensitivity. By revealing that CO_2_ circuitry is not only a relay but an amplified echo chamber, they have uncovered a key circuit strategy and opened the door to new questions about how the brain prioritizes crucial survival cues.

## Abbreviations

LN: local neuron
OSN: olfactory sensory neuron
PN: projection neurons
